# Analysis of early postoperative complications in patients with resectable rectal cancer after neo-adjuvant chemo-radiotherapy

**DOI:** 10.1186/1471-2482-13-S1-A25

**Published:** 2013-09-16

**Authors:** Luciano Grimaldi, Stefano Reggio, Mario Pannullo, Giovanni De Palma, Giovanni Aprea, Bruno Amato, Michele Danzi

**Affiliations:** 1 Department of Specialized Surgery, Division of Gastrointestinal Surgery Rehabilitation of Election and Emergency. “Federico II” University, Naples, Italy

## Background

Surgery is presently playing a leading role in rectal cancer treatment, but the natural history of the disease, featured by a high local recurrence rate (30-40% until the 80-90 years), suggested the possibility of using multimodal treatment regimen : radiotherapy (before, during and after surgery) possibly associated with chemotherapy [1,2]. This approach is recommended for the maority of patients with stage II (negative lymph nodes with tumor invading the muscularis) or with stage III (positive lymph nodes but without metastases after a time span). The neoadjuvant radio and chemo therapy, though having no impact on global survival rate, associated to TME reduce the local recurrence rate, provide a higher control level of systemic disease (micro metastases eradication), increase the rate of complete pathological responses, and allow a higher rate of “sphincter sparing” surgery [3,4].

## Methods

From August 2006 to March 2011, 50 patients affected by rectal cancer (adenocarcinoma, with lower margin ≤ 12 cm and the top margin ≤ 16 cm from the anal margin, T3-4 and / or N +, M0) were treated with chemotherapy (capecitabine) and radiotherapy (45 Gy) neo-adjuvant in our A.O.U. Federico II; each of them underwent to surgery after 5-10 weeks from the last CT-RT cycle completion. They were all studied by a multidisciplinary team (oncologists, radiotherapists, endoscopists and surgeons). Were excluded from our study all patients in state of pregnancy, breast feeding, with previous or present neoplasms, heart patients and patients with neuro-psychic disorders or active infections. We carried out a retrospective study in order to assess the possible difference in the occurrence of early post-operatory complications (within 30 days) in this group of 50 patients (Group A RT-CT neo-adjuvant), and another group of 35 patients treated in the past (even before 2006) with surgery only. For each of them was taken into account age, gender, ASA score, the surgery that was chosen, the tumor stage, its position and distance from anal margin, TRG (only for patients of Group A). The collected data were studied through 2x2 contingency tables and statistics of χ 2, the associations between the independent variables and complications were analyzed by calculating the Odds Ratio, p values <.05 were considered statistically significant.

## Results

The 2 groups were matched for age, gender, ASA, location and stage of the cancer, the type and duration of surgery, lenght of hospital stay. A kind of association was highlighted between neo adjuvant therapy and “sphincter sparing” surgery with p<.025. In A Group 78% of patients had a protective ileostomy as well, vs 42% of B Group. Although there were no statistically significant differences, the the rate of patients showing complicances is lower in A group (16 patients, 32%) with respect to B Group (14 patients, 40%). In both groups, urinary disorders are the most frequently observed (cystitis, urinary incontinence, bladder atony, hydronephrosis). The wound infection rate, pelvic abscess, dehiscence of the anastomosis, post-operative bleeding were similar in both groups; as well as with regard to sexual dysfunction (1 case of impotence erigendi in B group). Mortality rate, low in both groups, was of 1 case in A group and 1 case in B group, cases anyway connected to to co-morbidity factors which were pre-existing to surgery.

## Conclusions

Our study highlights that the application of a multimodal regimen to patients affected by rectal cancer , based on neo-adjuvant chemotherapy and radiotherapy followed by surgery after 5-10 weeks (TME) is not connected to a higher risk of early complications, and in particular does not cause any statistically significant increase in anastomotic dehiscence. But patient conditions before treatment (ASA III-IV), positive lymph nodes are independent risk factors for the development of complications, while the performing of a protective ileostomy reduces the severity and at least in part the incidence of anastomotic leakage.
